# Integrative data semantics through a model-enabled data stewardship

**DOI:** 10.1093/bioinformatics/btac375

**Published:** 2022-06-02

**Authors:** Philipp Wegner, Sebastian Schaaf, Mischa Uebachs, Daniel Domingo-Fernández, Yasamin Salimi, Stephan Gebel, Astghik Sargsyan, Colin Birkenbihl, Stephan Springstubbe, Thomas Klockgether, Juliane Fluck, Martin Hofmann-Apitius, Alpha Tom Kodamullil

**Affiliations:** Department of Bioinformatics, Fraunhofer Institute for Algorithms and Scientific Co mputing (SCAI), Sankt Augustin 53754, Germany; Department of Bioinformatics, Fraunhofer Institute for Algorithms and Scientific Co mputing (SCAI), Sankt Augustin 53754, Germany; Department of Neurology, University Hospital Bonn (UKB), Bonn 53127, Germany; Department of Bioinformatics, Fraunhofer Institute for Algorithms and Scientific Co mputing (SCAI), Sankt Augustin 53754, Germany; Enveda Biosciences, Boulder, CO 80301, USA; Department of Bioinformatics, Fraunhofer Institute for Algorithms and Scientific Co mputing (SCAI), Sankt Augustin 53754, Germany; Bonn-Aachen International Center for IT (B-IT), Rheinische Friedrich-Wilhelms-Universität Bonn, Bonn 53115, Germany; Department of Bioinformatics, Fraunhofer Institute for Algorithms and Scientific Co mputing (SCAI), Sankt Augustin 53754, Germany; Department of Bioinformatics, Fraunhofer Institute for Algorithms and Scientific Co mputing (SCAI), Sankt Augustin 53754, Germany; Bonn-Aachen International Center for IT (B-IT), Rheinische Friedrich-Wilhelms-Universität Bonn, Bonn 53115, Germany; Department of Bioinformatics, Fraunhofer Institute for Algorithms and Scientific Co mputing (SCAI), Sankt Augustin 53754, Germany; Bonn-Aachen International Center for IT (B-IT), Rheinische Friedrich-Wilhelms-Universität Bonn, Bonn 53115, Germany; Department of Bioinformatics, Fraunhofer Institute for Algorithms and Scientific Co mputing (SCAI), Sankt Augustin 53754, Germany; Department of Neurology, University Hospital Bonn (UKB), Bonn 53127, Germany; German Center for Neurodegenerative Diseases (DZNE), Bonn 53127, Germany; Department of Bioinformatics, Fraunhofer Institute for Algorithms and Scientific Co mputing (SCAI), Sankt Augustin 53754, Germany; Department Knowledge Management, Information Centre for Life Sciences (ZBMED), Bonn 53115, Germany; Department of Bioinformatics, Fraunhofer Institute for Algorithms and Scientific Co mputing (SCAI), Sankt Augustin 53754, Germany; Bonn-Aachen International Center for IT (B-IT), Rheinische Friedrich-Wilhelms-Universität Bonn, Bonn 53115, Germany; Department of Bioinformatics, Fraunhofer Institute for Algorithms and Scientific Co mputing (SCAI), Sankt Augustin 53754, Germany; Causality Biomodels, Kinfra Hi-Tech Park, Kalamassery, Cochin, Kerala 683503, India

## Abstract

**Motivation:**

The importance of clinical data in understanding the pathophysiology of complex disorders has prompted the launch of multiple initiatives designed to generate patient-level data from various modalities. While these studies can reveal important findings relevant to the disease, each study captures different yet complementary aspects and modalities which, when combined, generate a more comprehensive picture of disease etiology. However, achieving this requires a global integration of data across studies, which proves to be challenging given the lack of interoperability of cohort datasets.

**Results:**

Here, we present the Data Steward Tool (DST), an application that allows for semi-automatic semantic integration of clinical data into ontologies and global data models and data standards. We demonstrate the applicability of the tool in the field of dementia research by establishing a Clinical Data Model (CDM) in this domain. The CDM currently consists of 277 common variables covering demographics (e.g. age and gender), diagnostics, neuropsychological tests and biomarker measurements. The DST combined with this disease-specific data model shows how interoperability between multiple, heterogeneous dementia datasets can be achieved.

**Availability and implementation:**

The DST source code and Docker images are respectively available at https://github.com/SCAI-BIO/data-steward and https://hub.docker.com/r/phwegner/data-steward. Furthermore, the DST is hosted at https://data-steward.bio.scai.fraunhofer.de/data-steward.

**Supplementary information:**

Supplementary data are available at *Bioinformatics* online.

## 1 Introduction

Vast amounts of patient data of heterogeneous types are produced on a daily basis. This data can be generated in different locations, including hospitals or primary care centers, in regional multicenter studies, national centers and international consortia. Typically, while there are multiple studies that collect heterogeneous information from patients having a certain condition, designs of studies and records often do not match. Consequently, the underlying data cannot be directly compared across studies without a semantic harmonization that enables dataset interoperability. This lack of interoperability across datasets prevents one from getting the most out of the data as well as conducting independent validations on external datasets (Birkenbihl *et al.*, 2020).

A solution to this problem lies in the establishment of a so-called global clinical data model (CDM) (see examples in Supplementary Table S1) which aims at integrating information across heterogeneous datasets. In doing so, such global CDMs could be used by cataloging activities, repositories and computational data environments in order to manage data based on schemata and metadata templates, ultimately improving interoperability and exchange across resources. Individual research institutions also stand to benefit as they can organize data according to a common data model in their domain. As such, developing domain-specific common data models can have a catalyst effect that accelerates the sharing and exchange of data that can influence future design of studies. Additionally, existing common data models are not yet capable enough to map all variables from a particular topic, for instance, dementia (Supplementary Text S1).

Here, we present the Data Steward Tool (DST) which can be used to automatically standardize clinical datasets, map them to established ontologies, align them with OMOP standards (a widely used data model for health data), and export them to a FHIR-based format. To standardize specific variables from dementia which could not be mapped to existing ontologies or data standards, we developed a CDM in the context of dementia, built from various datasets—Alzheimer’s disease datasets [ADNI (Mueller *et al.*, 2005) and AddNeuroMed (Lovestone *et al.*, 2009)], routine clinical datasets (from University Hospitals Bonn and Aachen, Germany) and other datasets covering neurodegenerative diseases from DZNE (https://www.dzne.de) and EUROSCA (http://www.eurosca.org).

## 2 Implementation

We implemented a web application called the DST that allows users to read, edit and use the CDM to standardize real-world research data. The tool consists of RESTful APIs served by a Django application on a MongoDB database (technical details are described in Supplementary Text S2); thus, providing a user-friendly environment that makes the underlying data accessible to the scientific community.

To demonstrate the DST, we accessed and investigated major dementia studies (Supplementary Text S3) and identified the variables they shared in common. We found that the majority of these variables comprised patient information, such as age, sex or initial diagnosis, biomarker measurements, as well as measurements from neuropsychological tests, such as the Boston Naming Test. This set of variables (Supplementary Table S2) was then used as the groundwork to extend the data model as we gained access to new studies by connecting the variables of additional studies to equivalent variables present in the CDM (Supplementary Text S4). Finally, we extracted data types and value ranges for each of the variables and assigned every variable to a specific data modality.

## 3 Results

### 3.1 Data Steward Tool for data standardization

**Fig. 1.**
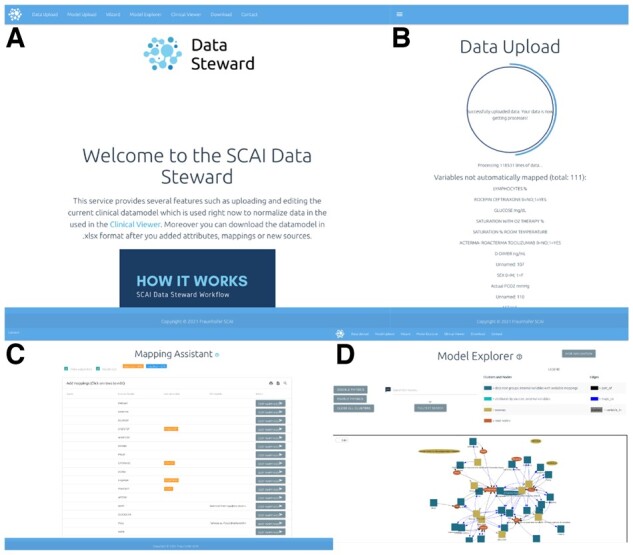
Overview of Data Steward Tool features. (**A**) Home page. (**B**) Data upload page. (**C**) Mapping assistant view. (**D**) Data Model explorer view

We developed a web application called the DST that provides an interface to visualize the model, extend it with new variables, add mappings and read clinical data through a user-friendly interface (Fig. 1A). Furthermore, the DST is capable of automatically mapping external variables onto the CDM through fuzzy string matching (Supplementary Text S5). Performing full-text searches throughout the entire data model grants the possibility of using the system as a searchable variable catalog for dementia research. Additionally, uploading clinical data onto the system allows for data standardization and the storage of harmonized data in a central data repository (Fig. 1B) The uploaded standardized data can be queried via a RESTful API. Through these APIs, users can access the content of the DST using their own tools (e.g. Jupyter notebook). Uploading and standardizing data is a guided multi-step process (detailed description in Supplementary Text S6), where the user begins by loading the data, often a 2D data table, to an interface via drag and drop and is then given the opportunity to manually map variables that were not found in the current data model (Fig. 1C). During this process, the user is supported with autosuggestions to automatically add terms from OLS (Ontology Lookup Service) if no suitable variable could be found. Finally, the DST provides a graph-based view (Fig. 1D) of the model where the user can interactively explore the entirety of the model.

To make the DST fully compatible with existing approaches currently used in industry, the tool functions as a FHIR node that is capable of providing patient data in FHIR format. Furthermore, the data model is aligned with OMOP (Supplementary Text S7) and can easily be enhanced with existing data models (Supplementary Text S8).

### 3.2 Clinical data model for dementia

The CDM consists of 277 common variables representing cohort demographic information, various clinical assessments and further dementia-related biomarkers. The CDM was designed to store metadata about the variables themselves, including definitions, data type and value ranges as well as information about the variable names already mapped onto it. The CDM can constantly and rapidly expand as more studies are mapped to it. By enriching the variables present in the data model with mappings to other resources, the system is capable of standardizing data from different origins (Supplementary Text S9). Finally, while the data model is stored in a database, it can be exported to OWL (Web Ontology Language), RDF (Resource Description Framework) as well as tabular formats such as excel and csv.

### 3.3 Clinical viewer

The DST is accompanied with the Clinical Viewer (https://data-steward.bio.scai.fraunhofer.de/), a complementary visualization tool to explore the clinical data stored in the DST using various visualization techniques such as scatter plots, pie charts and histograms. In order to demonstrate the DST and the Clinical Viewer, we used publicly available patient data to generate 10 virtual patients similar to those of the ADNI dataset (Mueller *et al.*, 2005). These patients were then duplicated until we had datasets of 1000 and 10 000 patients (see details in Supplementary Text S10). The synthetic 1000 patients ADNI dataset (https://github.com/phwegner/synADNI/tree/main/output) is currently used as an example in the demo instances of the DST (Supplementary Text S11).

## 4 Discussion and future work

The Data Steward Tool is a web application designed to bring a convenient and easy-to-use solution for the semantic integration of clinical data. By design, the system’s backend functions as a generic solution for data standardization and provides the possibility to map cohort datasets to global data models and ontologies. Moreover, the DST has various applications like the Clinical Viewer or could act as the data pre-processor for machine learning tasks. Any new dataset can be described and qualified in relation to the common data model, making it instantly clear what the strengths and weaknesses of a dataset are. For example, a common CDM would implicitly allow for an assessment of the coverage (with respect to variables) of a new study by mapping the new study to the common data model. As further steps, we plan to enrich the DST with more complex query APIs to make the tool usable for future data science tasks in bioinformatics. In order to further improve the data model, we plan to add more variables and mappings from diverse data sources and clinical studies. Finally, we aim to establish value transformations in order to enable the DST handling more complex mappings that require some sort of transformation between certain value ranges or units, plus an extension so that the DST can capture more complex mappings such as the relation between height or weight and body mass index.

## Funding 

This work has been supported by the IDSN project (project number: 031L0029 [A-C]).

*Conflict of Interest:* D.D.-F. received salary from Enveda Biosciences.

## Supplementary Material

btac375_Supplementary_Data
