# Correction to: Integrative data semantics through a model-enabled data stewardship

**DOI:** 10.1093/bioinformatics/btac635

**Published:** 2022-09-23

This is a correction to: Philipp Wegner, Sebastian Schaaf, Mischa Uebachs, Daniel Domingo-Fernández, Yasamin Salimi, Stephan Gebel, Astghik Sargsyan, Colin Birkenbihl, Stephan Springstubbe, Thomas Klockgether, Juliane Fluck, Martin Hofmann-Apitius, Alpha Tom Kodamullil, Integrative data semantics through a model-enabled data stewardship, *Bioinformatics*, Volume 38, Issue 15, 1 August 2022, Pages 3850–3852, https://doi.org/10.1093/bioinformatics/btac375

In the originally published version of the manuscript, there was an error in the Abstract. In the “Availability and implementation” section, the link to where the DST is hosted should read https://data-steward.bio.scai.fraunhofer.de/data-steward instead of https://data-steward.bio.sca.fraunhofer.de/data-steward. This error has been corrected online.

