# Supplementary material for: Integrative data semantics through a model-enabled data stewardship
Source: Bioinformatics. 2022 Jun 2;38(15):3850–2. doi: 10.1093/bioinformatics/btac375 (PMC9344835; doi:10.1093/bioinformatics/btac375)
Supplement: btac375_Supplementary_Data [file btac375_supplementary_data.pdf]

# Supplementary File

## Supplementary Tables

| Name | Description | Source |
|---|---|---|
| OMOP | Data model for observational health data | https://www.ohdsi.org/data-standardization/ |
| FHIR | **F**ast **H**ealthcare **I**nteroperability **R**esources is a standard for health care data exchange | https://www.hl7.org/fhir/overview.html |
| Ga4GH | GA4GH provides a tool kit for standardized genomic data sharing | https://ga4gh.github.io/tool-registry-service-schemas/DataModel/ |
| I2b2 | i2b2 provides software for structured integration / normalization of health data | https://www.i2b2.org/ |

**Supplementary Table 1. Global data models.** Examples of global clinical data models.

| Variable name | Short description |
|---|---|
| SEX | Gender |
| APOE_STATUS | ApoE Risk group |
| DOB | Date of birth |
| RW_BNTTOT | Raw score of Boston Naming Test |
| RW_CERCP | Constructive Practice - direct (raw score) |
| RW_CERCPR | Constructive Practice - delayed (raw score) |
| RW_CERCPSAV | Constructive Practice - Savings (raw score) |
| RW_CERDISC | Word list - Discriminability (raw score) |
| RW_CERDRLCT | Word list - Retrieval after interference (raw score) |
| RW_CERDRLIT | Word list - Intrusions (Raw Score) |
| RW_CERRL | Word list - sum (raw value) |
| RW_CERRL1CT | Word list - 1st round (raw score) |
| RW_CERRL2CT | Word list - 2nd round (raw score) |
| RW_CERRL3CT | Word list - 3rd round (raw score) |
| RW_CERWLSAV | Word list - Savings (raw value) |
| RW_MMSTOT | Mini Mental State Test (raw score) |
| RW_PFSTOT | Verbal fluency phonetically (raw score) |
| RW_TMTA | TMT Boy Scout Test - Part A (raw score) |
| RW_TMTB | TMT Boy Scout Test - Part B (raw score) |
| RW_TMTBA | TMT Boy Scout Test - B / A (raw score) |
| RW_VFATOT | Verbal fluency cat. (Raw value) |
| LIQ_A40_RESN | CSF Amyloid beta 40 |
| LIQ_A42_A40_RATIO | CSF Ratio 42/40 |
| LIQ_A42_RESN | CSF Amyloid beta 42 |

| | |
|---|---|
| LIQ_LEU_RESN | CSF leukocytes |
| LIQ_PTP_RESN | CSF pTau |
| LIQ_TP_C_RESN | CSF Protein (total) |
| LIQ_TTP_RESN | CSF Tau (total) |
| LIQ_A38_RESN | CSF Amyloid beta 38 |
| MEMORY | Memory impairment |
| LANGUAGE | Speech disorder |
| DIAGNOSIS | Diagnosis |
| LIQ_A42_A40_RATIO_CALC | CSF Ratio 42/40 |
| SCACAT | Patient category (SCA known, SCA unknown, control) |
| SCACAT2 | SCA genotype |
| CARRIER | Mutation carrier vs. first-degree relative of mutation carrier |
| ATAXIC | Ataxic yes / no |
| AOO | Age at first gait disorder (age of onset) |
| MUTATION | Repeat or other mutation |
| SHORT | Number of CAG repeats normal allele |
| LONG | Number of CAG repeats longer allele |
| MUTATIONSPC | Specify mutation |
| TESTED | Tested relative |
| SCANCAT | Genotypes excluded from SCA unknown |
| ATAXIA | With SCA unknown ataxic yes / no |
| AOO2 | At SCA unknown AOO |
| SCACONTROL | Control group membership (family, community) |
| OLDSTUDY | Previous study |
| OLDID | Previous ID |
| HANDEDNESS | Handedness |
| SGAIT | Score SARA item Gait |
| SSTANCE | Score SARA item stance |
| SSITTING | Score SARA item sitting |
| SDISTURB | Score SARA item speech |
| SCHASERI | Subscore right SARA item finger chase |
| SCHASELE | Subscore left SARA item finger chase |
| SFINGERMEAN | Score SARA item finger chase |
| SFINGERRI | Subscore right SARA item nosefinger |
| SFINGERLE | Subscore left SARA item nosefinger |
| SFINGERNOSEMEAN | Score SARA item nosefinger |
| ALHANDRI | Subscore right SARA item diadochokinese |
| ALHANDLE | Subscore left SARA item diadochokinese |
| SALTHANDMEAN | Score SARA item diadochokinese |
| SHEELRI | Subscore right SARA item heelshin |
| SHEELLE | Subscore left SARA item heelshin |
| SHEELSHINMEAN | Score SARA item heelshin |
| SARASUM | SARA sum score |

**Supplementary Table 2. Variables used as a base set.**

# Supplementary Text

## Supplementary Text 1. Reasons outlining why OMOP was not suitable for dementia datasets

The OMOP Data Model, developed and maintained by **OHDSI**, is highly popular in bioinformatics since it provides a good standardization capability, while OHDSI also offers analysis tools that work with data in OMOP.

Since the beginning of the project, we have worked very closely with clinicians from the University Clinic of Bonn and the DZNE. During that time, we had insight into multiple different studies dealing with dementia and ataxia data. There was no common structure between those data sources and we quickly realized that in order to harmonize as much study data as possible we need a very flexible data model. Since we are working with data from one specific field (neurodegenerative diseases), we decided to go for a custom data model that does not come with the overhead of a data model that tries to capture all health observation data like OMOP does. Additionally there are some variables in the datasets we had access to that were not in OMOP and nor were there equivalent variables in OMOP. Such examples are :

- Neurological test questions like FAQFORM, FAQFINAN, FAQSHOP, etc. from the DZNE datasets
- Ataxia related variables like SGAIT, SSTANCE, SSITTING, etc
- ENTORHINAL_ICV, FUSIFORM_ICV, etc. from ADNI

## Supplementary Text 2. Implementation of the Data Steward Tool

Django is a high level web-framework written in Python that offers plugins for MongoDB as well as for providing RESTful APIs. The application holds the structure of the Clinical Data Model in terms of Python classes and is capable of communicating that to the underlying database. Uploaded data as well as mappings and the complete data logic is handled here. Moreover the application provides the APIs that can be queried via other systems. The Vue.js web application uses that API to yield a visual interface. Vue.js is a progressive Javascript framework to build single page applications like the Data Steward Tool. The communication between the Vue.js app and the Django backend is based on the RESTful API principle that uses JSON as notation for data exchange. The underlying MongoDB database stores the underlying data model with all its variables and mappings as well as normalized clinical data uploaded by the user. The deployment of the services is realized in a microservice architecture with Docker.

## Supplementary Text 3. Resources used for developing CDM

During the development of the Clinical Data Model we used different data sources in the form of clinical studies or patient registries. Most of the base variables are taken from, or influenced by,

two major studies from the German Center for Neurodegenerative Diseases (DZNE). Those two studies are DESCRIBE DZNE - Clinical Register Study of neurodegenerative Disorders[1] and DELCODE DZNE - Longitudinal Cognitive Impairment and Dementia study[2]. Additionally, we investigated international studies like Alzheimer's Disease Neuroimaging Initiative: ADNI[3] in order to be aligned with variables and terms used outside of the German research landscape. Other studies and (meta-) data resources that were used either during the development of the base variable set or in the later work to establish mappings were AddNeuroMed - the European collaboration for the discovery of novel biomarkers for Alzheimer's disease[4], terms from the Diagnostic and treatment center for memory disorders (DBGA) of UKB as well as multiple studies related to Ataxia we got access to from the DZNE: ESMI (European Spinocerebellar Ataxia Type 3/Machado-Joseph Disease Initiative)[5], SCA Registry (Registry for Spinocerebellar Ataxias (SCA))[6].

## Supplementary Text 4. Definition: Variable Mappings

A crucial part of the data model are variable mappings. The most common definition for that is a reference from one variable VAR1 to another one VAR2, where VAR1 and VAR2 are semantically equivalent. A single mapping in the CDM holds the information about the external variable VAR1 and the internal variable VAR2 plus the source of VAR1. All variable mappings can be viewed in tabular form: https://data-steward.bio.scai.fraunhofer.de/data-steward/table. With more and more mappings the system can read and understand data from many different data sources. In the future we expect the number of mappings (currently 276) to outgrow the number of internal variables (277) by far.

## Supplementary Text 5. Fuzzy Matching

Fuzzy string matching in the context of the DST means that we assign one variable to another based on their string similarity (https://en.wikipedia.org/wiki/Edit_distance ). The success of the fuzzy string matching highly depends on the variable naming since ABETA 42 → abeta_42 is easy to be mapped based on string similarity but things like gender → sex will most certainly never be mapped by this approach. Future work includes inproving the mapping assistant with AI-based entity matching.

[1] https://www.dzne.de/en/research/studies/clinical-studies/describe-1-1/
[2] https://www.dzne.de/en/research/studies/clinical-studies/delcode/
[3] http://adni.loni.usc.edu/
[4] https://pubmed.ncbi.nlm.nih.gov/19906259/
[5] https://www.dzne.de/en/research/studies/clinical-studies/esmi/
[6] https://www.dzne.de/en/research/studies/clinical-studies/sca-registry/

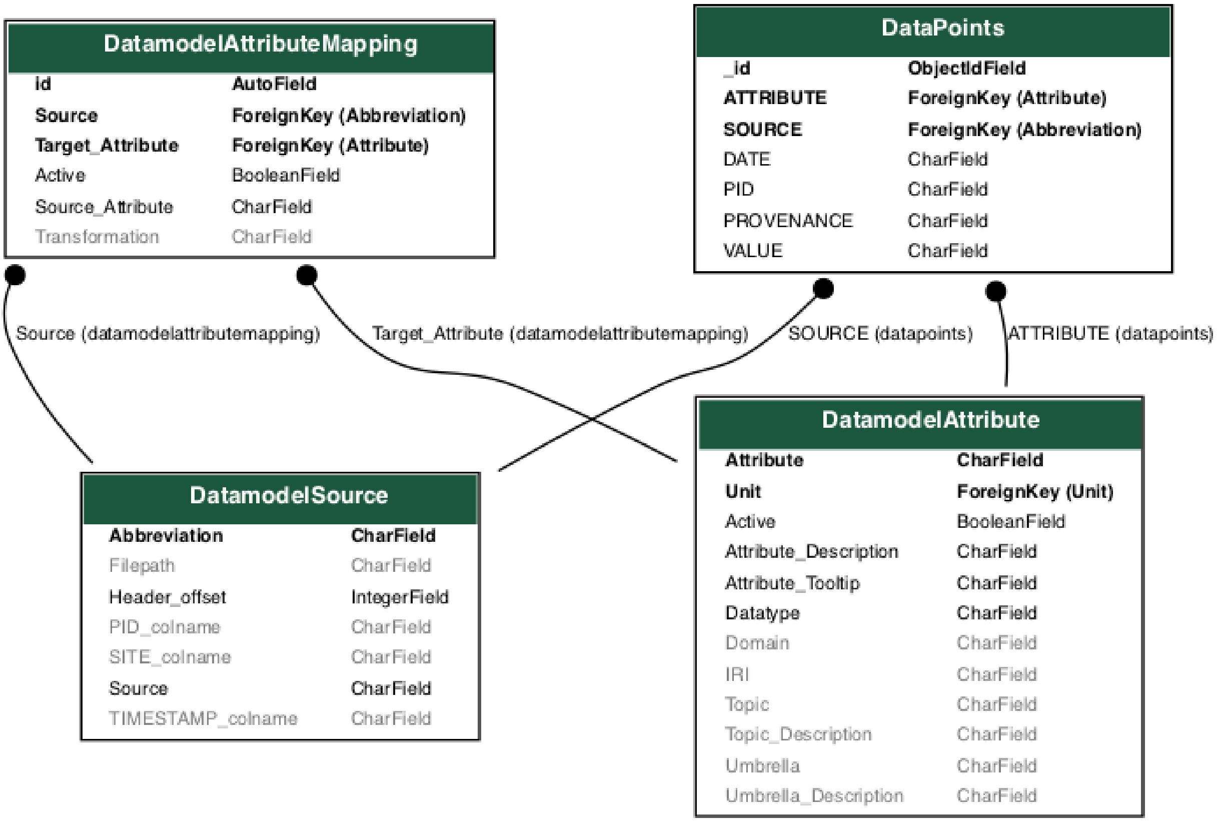


**Supplementary Figure 1. UML Diagram of CDM on Database level**

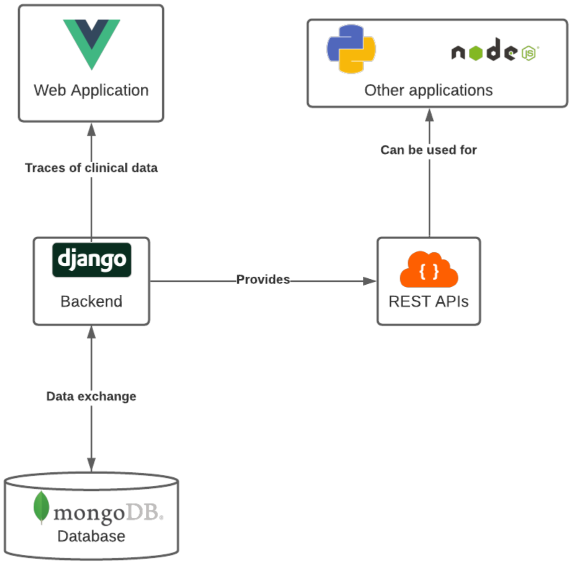


**Supplementary Figure 2. Interactions of the different Components of the DS.**

## Supplementary Text 6. Tutorial of the upload process

Clinical data is typically stored in 2D data tables, where in each row one measurement for one patient or subject is represented with the variable name and the measured value. The tool reads data in a csv-like format where each line represents one measurement. For example one line consists of the patient's id (Entity), the variable AGE (Attribute), and the patient's age (Value). A software transforming clinical data, that has multiple values per row, into the correct format is available. Such representation of clinical data is called EAV (**E**ntity **A**ttribute **V**alue) format. The Data Steward Tool can read and process those EAV files in a drag and drop section (**Figure 3**).

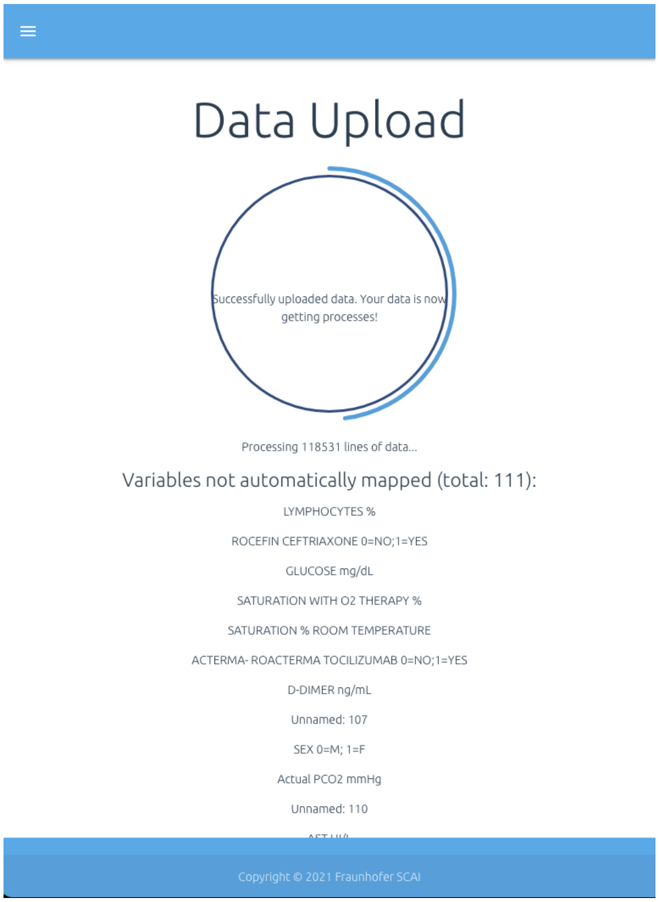


**Supplementary Figure 3**. **Data upload screenshot**

During the reading process, the user is updated with live feedback on how many lines in the file were found for how many distinct patients and what variables could not be found in the model. After finishing the process, the tool yields a summary of the results for the user to analyze. In the case that every variable was found in the underlying Data Model, everything is done and the data is successfully semantically integrated. If that's not the case the user can always map the variables onto CDM via a guided process. From the upload feedback, the user can go to the mapping assistant (**Figure 4**).

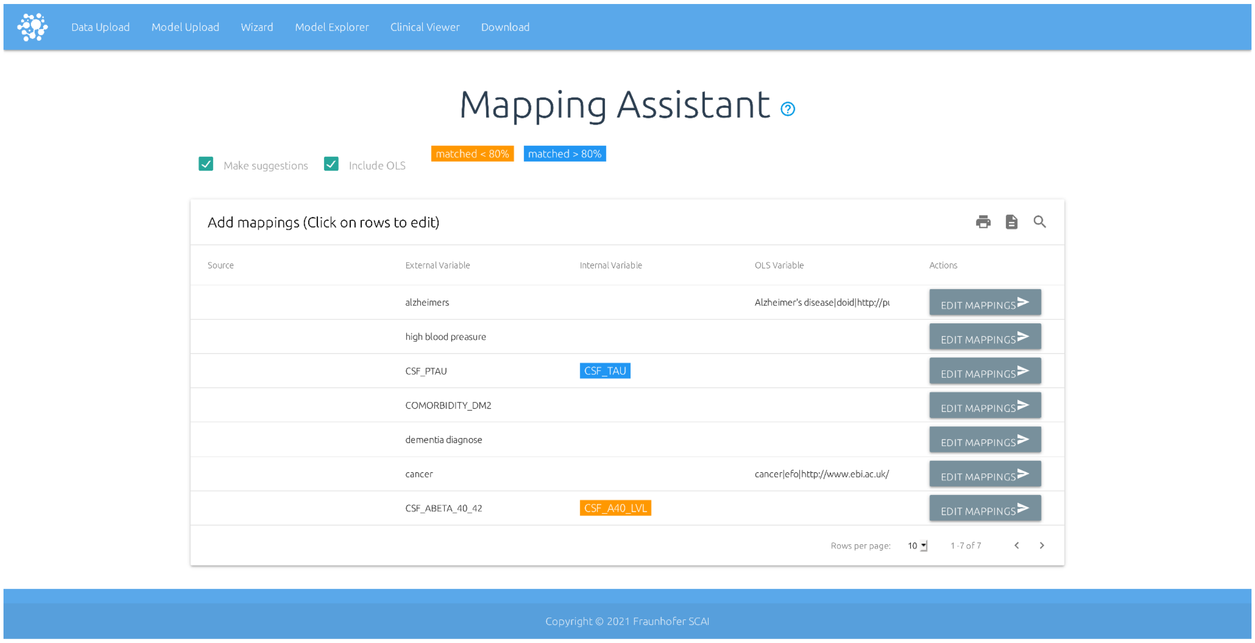


**Supplementary Figure 4. Mapping Assistant Screenshot.**

The assistant view is a table view where each line is an unmapped variable. The table comes with autocomplete and auto-suggestion features based on nearest neighbor search with edit distance as well as string comparison of variables and variable definition texts. Moreover, the user can

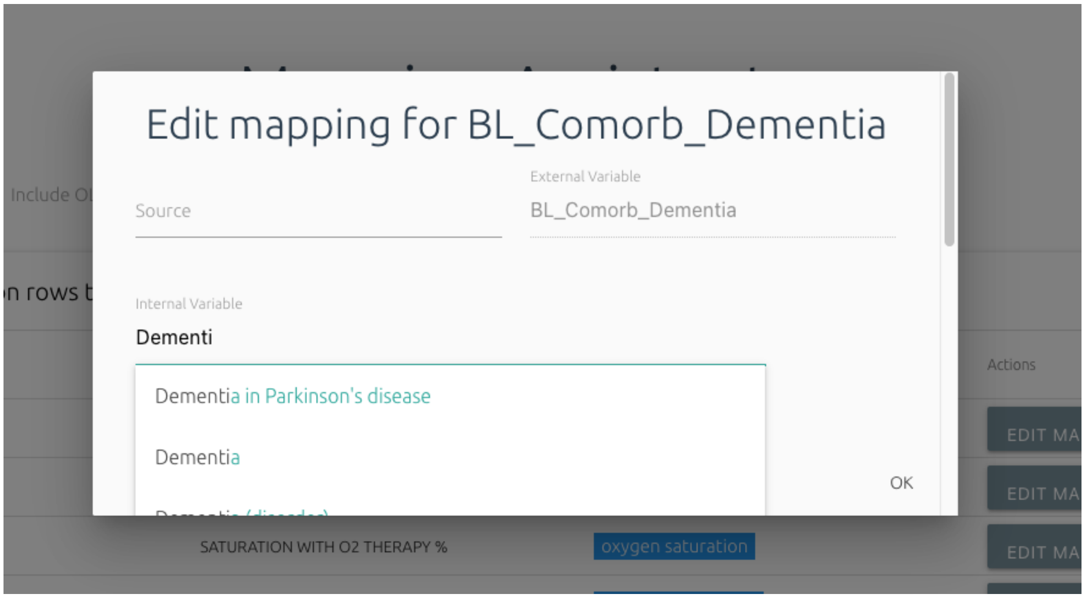


**Supplementary Figure 5. Edit a Mapping.**

activate the OLS (**O**ntology **L**ookup **S**ervice) autocomplete to conveniently integrate terms from major ontologies if no suitable variable is present in the current model. If a suitable variable was

found the user has to select a source for the external variable (e.g. ADNI) and then the mapping can be submitted and the table gets reduced by the respective line. In the case that the mapping assistant suggests a wrong mapping you can edit the respective line manually via a dialog window, backed up by autocompletion (**Figure 5**). Thus, the user has a comfortable way to map all of their data onto the CDM.

## Supplementary Text 7. Aligning CDM with FHIR and OMOP

In bioinformatics, a very crucial part of research is exchanging health data between healthcare ecosystems or institutions. The FHIR (**F**ast **H**ealthcare **I**nteroperability **R**esources) standard of HL7 is a common solution to do that. Hence the Data Steward Tool can work as a FHIR server[7] by providing multiple APIs (API Documentation[8]) that return either all patient data in FHIR format (observation resource defined by FHIR standard[9]) or single measurements for one patient. Moreover users can query the DS for certain patients in certain studies (patient resource[10]).

As mentioned above, the OMOP data model is an established solution for data standardization in bioinformatics and hence it is inevitable that the CDM is aligned with OMOP. Thus, we found mappings between every variable of the CDM and the OMOP standard vocabulary using OHDSI's Athena browser[11]. This embedding of the CDM onto OMOP is updated regularly if the CDM changes or after a certain time to keep the mappings up to date. Up to now, we were able to map 181 variables out of CDM to the OMOP standard vocabulary.

## Supplementary Text 8. Importing other data models by the example of i2b2

As mentioned in the paper there are some major data models out there that aim to capture general biomedical data like OMOP or domain specific data like GA4GH. The i2b2 data model is capable of describing general clinical data. In order to contribute to a research landscape where data interoperability is crucial it is important that the data steward tool is able to function with other data models in its backend. We have created a python package available on GitHub[12] that contains an example of how to import all i2b2 variables into the DST.

## Supplementary Text 9. Standardizing dementia data with the CDM

With the Data Steward Tool we were able to standardize mutiple dementia data sources and map them to one common data model the CDM. Those sources were among others DESCRIBE (https://www.dzne.de/en/research/studies/clinical-studies/describe-1-1/) and DELCODE (https://www.dzne.de/en/research/studies/clinical-studies/delcode/) as well as other data sources we had access to from the University Clinic Bonn (UKB).

---

[7] At the moment we only provide GET Apis export data to the FHIR format
[8] https://data-steward.bio.scai.fraunhofer.de/data-steward/swagger
[9] https://www.hl7.org/fhir/observation.html
[10] https://www.hl7.org/fhir/patient.html
[11] https://athena.ohdsi.org/search-terms/start
[12] https://github.com/phwegner/DST-API

## Supplementary Text 10. Duplicating virtual patients

We manually generated 10 virtual patients from ADNI data using a mixed technique consisting of statistical bootstrapping and adding normal distributed noise to the observation values. The resulting data, and with that the whole method, was evaluated and tested using the currently known data ranges from the original 'true' ADNI data from the study. By that, we could generate artificial data that we could use for public instances without any privacy concerns while also demonstrating realistic data values. Results and implementations are available on GitHub[13].

## Supplementary Text 11. Using the virtual cohort

In a public GitHub Repo[14] we provide the 1000 patients virtual cohort eav file. This can directly be read by the DST. You can upload it here: https://data-steward.bio.scai.fraunhofer.de/data-steward/datapoints and the upload process.

[13] https://github.com/phwegner/AAD_N_DR
[14] https://github.com/phwegner/synADNI/tree/main/output
